# Supplementary material for: Generative AI and academic scientists in US universities: Perception, experience, and adoption intentions
Source: PLoS One. 2025 Aug 28;20(8):e0330416. doi: 10.1371/journal.pone.0330416 (PMC12393709; doi:10.1371/journal.pone.0330416)
Supplement: S1 Appendix — (DOCX) [file pone.0330416.s001.docx]

**S1 Appendix. Non-response bias analysis**

We evaluated potential nonresponse bias in our survey sample with two sets of analyses. These examined whether our respondent sample was significantly different across three demographic characteristics (i.e., gender, academic field, and rank) from (a) the initial sample frame used for recruiting SciOPS panel members, and (b) the sample of SciOPS panel members randomly selected as the sample for this survey.

Table S1 shows the first set of results, which examines demographic differences between survey respondents (n=232) and the initial sample frame of academic scientists in all six fields originally employed to recruit the SciOPS panel (n=18,504). T-test results indicated no differences between survey respondents and the initial sample frame in terms of gender representation. There were also no observed differences by academic rank. Academic scientists in two academic fields were significantly underrepresented in the final sample of survey respondents, compared with the academic field composition of the initial sample used for SciOPS panel recruitment. These included biologists (33.7% of initial sample frame for SciOPS panel recruitment vs. 25.4% of survey respondents, p-value < 0.005) and public health academic scientists (18.4% of initial sample frame for SciOPS panel recruitment vs. 8.6% of survey respondents, p-value < 0.005). Civil and environmental engineers were significantly overrepresented in the final sample of survey respondents (14.5% of the initial sample frame for SciOPS panel recruitment vs. 22.0% of survey respondents, p-value < 0.01).

**Table S1**. T-test results for comparison of demographic composition of survey respondents and initial SciOPS panel recruitment frame.

| Construct | Variable | Initial Panel Recruitment sample (%) | Survey Respondents (%) | Group Differences (%) | P-value |
| --- | --- | --- | --- | --- | --- |
| Gender | Female | 32.8 | 35.3 | 2.5 | 0.419 |
| Field | Biology | 33.7 | 25.4 | 8.3*** | 0.004 |
|  | Chemistry | 13.1 | 15.9 | 0.24 | 0.235 |
|  | Computer and Information Science Engineering | 15.6 | 13.8 | 1.8 | 0.435 |
|  | Civil and Environmental Engineering | 14.5 | 22.0 | 7.5** | 0.006 |
|  | Geography | 4.7 | 14.2 | 9.5*** | 0.000 |
|  | Public Health | 18.4 | 8.6 | 9.8*** | 0.000 |
| Rank | Full Professor | 41.4 | 40.1 | 1.3 | 0.683 |
|  | Associate Professor | 20.8 | 19.0 | 1.8 | 0.486 |
|  | Assistant Professor | 20.7 | 21.6 | 0.9 | 0.747 |
|  | Non-tenure Track Researcher | 17.1 | 19.4 | 3.8 | 0.389 |
| (n) |  | (18504) | (232) |  |  |

* P <0.05, ** p < 0.01, *** p < 0.005

Table S2 shows the results of an additional set of t-test analyses which compared the demographic composition of those SciOPS panel members invited to participate in this survey (n=777) and those academic scientists who agreed to participate (n=232). There were no significant demographic differences by gender, academic field, or academic rank between these two groups.

**Table S2**. T-test results for comparison of demographic composition of survey respondents and sample of SciOPS participants.

| Construct | Variable | SciOPS Panel Members (%) | Survey  Respondents (%) | Group Differences (%) | P-value |
| --- | --- | --- | --- | --- | --- |
| Gender | Female | 35.6 | 35.3 | 0.3 | 0.940 |
| Field | Biology | 23.3 | 25.4 | 2.1 | 0.510 |
|  | Chemistry | 16.9 | 15.9 | 1.0 | 0.741 |
|  | Computer and Information Science Engineering | 12.0 | 13.8 | 1.8 | 0.475 |
|  | Civil and Environmental Engineering | 26.5 | 22.0 | 4.5 | 0.152 |
|  | Geography | 13.1 | 14.2 | 1.1 | 0.673 |
|  | Public Health | 8.2 | 8.6 | 0.4 | 0.855 |
| Rank | Full Professor | 40.1 | 40.1 | 0 | 0.985 |
|  | Associate Professor | 19.0 | 19.0 | 0 | 0.978 |
|  | Assistant Professor | 21.8 | 21.6 | 0.2 | 0.949 |
|  | Non-tenure Track Researcher | 19.0 | 19.4 | 0.4 | 0.906 |
| (n) |  | (777) | (232) |  |  |

* P <0.05, ** p < 0.01, *** p < 0.005
