# Supplementary material for: Generative AI and academic scientists in US universities: Perception, experience, and adoption intentions
Source: PLoS One. 2025 Aug 28;20(8):e0330416. doi: 10.1371/journal.pone.0330416 (PMC12393709; doi:10.1371/journal.pone.0330416)
Supplement: S2 Appendix — (DOCX) [file pone.0330416.s002.docx]

**S2 Appendix. Number of randomly selected institutions for sampling scientists**

**Table S3.** Number of randomly selected institutions for sampling scientists

| **Fields** | **Number of randomly selected R1 institutions** | **Number of all R1 institutions** |
| --- | --- | --- |
| Biology | 106 | 131 |
| Civil and environmental engineering | 46 | 131 |
| Geography | 46 | 131 |
| Public health | 61 | 61 |
| Computer and information science engineering | 45 | 131 |
| Chemistry | 62 | 131 |
