# Supplementary material for: Generative AI and academic scientists in US universities: Perception, experience, and adoption intentions
Source: PLoS One. 2025 Aug 28;20(8):e0330416. doi: 10.1371/journal.pone.0330416 (PMC12393709; doi:10.1371/journal.pone.0330416)
Supplement: S3 Appendix — (DOCX) [file pone.0330416.s003.docx]

**S3 Appendix. Survey instrument: Generative AI uses and impacts in academic research and education**

**Landing page**

**Welcome to SciOPS (Scientist Opinion Panel Survey)**

Thank you for being a member of [SciOPS](https://urldefense.com/v3/__https:/sci-ops.org__;!!IKRxdwAv5BmarQ!arez-_ywk453-ZvN8vDubHFkyz4sABBh9oqMhow364kgox4xqtMt8R-YX8Lt-UufTW8qTWDAD9-ZBTaO-pc$), a nationally representative panel of the Science, Technology and Innovation (STI) community.

We are interested in your personal views regarding **the use of generative artificial intelligence (AI) in academic research and education**. Thank you for your willingness to participate.

**Consent page**

**Welcome to SciOPS (Scientist Opinion Panel Survey)**

Thank you for being a member of [SciOPS](http://sci-ops.org/), a nationally representative panel of the Science, Technology and Innovation (STI) community. We are interested in your thoughts regarding generative artificial intelligence (AI) uses and impacts in academic research and education. Thank you for your willingness to participate.

All information collected is confidential and for comparative purposes only. Any data collected will only be reported as de-identified and shared in aggregate form with other researchers. This survey should take about 10 minutes to complete. By clicking “Start survey” you are providing consent to participate in this survey.

Participants must be 18 and older. If there is anything about the study or your participation that is unclear or that you do not understand, or if you have questions, you may contact Dr. Eric Welch, [EricWelch@asu.edu](mailto:EricWelch@asu.edu).

If you have any questions about your rights as a subject/participant in this research, you may email research.integrity@asu.edu or contact the Chair of the Human Subjects Institutional Review Board, through the ASU Office of Research Integrity and Assurance, at (480) 965-6788.

**Survey instrument**

**Section 1: Looking Ahead**

**We would like to begin by asking about your general opinions on some of the changes occurring in the world and how you believe they will impact our lives.**

*[s1_q1]* Thinking about the next few years, how much do you think each of the following will change the way we live in the United States? [The order of items *[s1_q1_a] to [s1_q1_g]* is randomized]

(1 Not at all, 2 Not very much, 3 Somewhat, 4 A lot)

*[s1_q1_a]* The spread of infectious diseases

*[s1_q1_b]* Artificial intelligence

*[s1_q1_c]* Climate change

*[s1_q1_d]* Political polarization

*[s1_q1_e]* Fusion energy

*[s1_q1_f]* Global conflicts

*[s1_q1_g]* Resource scarcity

*[s1_q1_h]* Other (please specify): *[s1_q1_other]*

**Section 2: Your perspectives on Generative AI generally**

**Now we would like to ask you more specifically about your perspectives on generative artificial intelligence (AI) and its potential impact on society.**

We refer to generative AI as a system that, in response to prompts written in natural language, dynamically generates content that is, or appears to be, novel.

*[Section2_Q2]* Does the increased use of generative AI in daily life make you feel______?

1 More excited than concerned

2 Equally excited and concerned

3 More concerned than excited

*[Section2_Q3]* How concerned are you about the following potential threats that may be posed by artificial intelligence? [Items order is randomized]

(1 Not at all concerned, 2 Not very concerned, 3 Somewhat concerned, 4 Very concerned, 5 Extremely concerned)

*[Section2_Q3_a]* Misinformation

*[Section2_Q3_b]* Over-reliance on it

*[Section2_Q3_c]* Loss of jobs

*[Section2_Q3_d]* Loss of creativity

*[Section2_Q3_e]* Threat to cyber security

*[Section2_Q3_f]* Loss of privacy

*[Section2_Q4]* Would you favor or oppose having a federal agency regulate the use of generative AI similar to how the FDA (Food and Drug Administration) regulates the approval of drugs and medical devices?

(1 Strongly support, 2 Somewhat support, 3 Neither support nor oppose, 4 Somewhat oppose, 5 Strongly oppose)

*Randomization:* Respondents are randomized for different treatment groups for the following set of questions *[Section2_Q5].*

If *[randomsetupsection2q5]=1* then show *[Section2_Q5_NoTreatment]* but skip *[Section2_Q5_TreatmentOnlyOption], [Section2_Q5_TreatmentOnlyQuestion], and [Section2_Q5_TreatmentBoth].*

If *[randomsetupsection2q5]=2,* then show *[Section2_Q5_TreatmentOnlyOption]* but skip *[Section2_Q5_NoTreatment], [Section2_Q5_TreatmentOnlyQuestion], and [Section2_Q5_TreatmentBoth].*

If *[randomsetupsection2q5]=3,* then show *[Section2_Q5_TreatmentOnlyQuestion]*, but skip *[Section2_Q5_NoTreatment], [Section2_Q5_TreatmentOnlyOption], and [Section2_Q5_TreatmentBoth].*

If *[randomsetupsection2q5]=4,* then show *[Section2_Q5_TreatmentBoth]*, but skip *[Section2_Q5_NoTreatment], [Section2_Q5_TreatmentOnlyOption], and [Section2_Q5_TreatmentOnlyQuestion].*

*[Section2_Q5_NoTreatment]* Which of the following is closest to your current point of view about the role of the government in regulating generative AI?

1 Federal government should not regulate generative AI technology, leaving its regulation to private industry.

2 Federal government should ban generative AI deployment until comprehensive research on its potential consequences is conducted.

*[Section2_Q5_TreatmentOnlyOption]* Which of the following is closest to your current point of view about the role of the government in regulating generative AI?

1 Federal government should not regulate generative AI technology, leaving its regulation to private industry.

2 Federal government should regulate generative AI technology, however, it should momentarily step back from doing so until comprehensive research on its potential consequences is conducted.

3 Federal government should ban generative AI deployment until comprehensive research on its potential consequences is conducted.

*[Section2_Q5_TreatmentOnlyQuestion]* Considering the possible ethical and unintended consequences generative AI may have, which of the following is closest to your current point of view about the role of the government in regulating generative AI?

1 Federal government should not regulate generative AI technology, leaving its regulation to private industry.

2 Federal government should ban generative AI deployment until comprehensive research on its potential consequences is conducted.

*[Section2_Q5_TreatmentBoth]* Considering the possible ethical and unintended consequences generative AI may have, which of the following is closest to your current point of view about the role of the government in regulating generative AI?

1 Federal government should not regulate generative AI technology, leaving its regulation to private industry.

2 Federal government should regulate generative AI technology, however, it should momentarily step back from doing so until comprehensive research on its potential consequences is conducted.

3 Federal government should ban generative AI deployment until comprehensive research on its potential consequences is conducted.

*[Section2_Q6]* How much do you support or oppose using generative AI for each of the following purposes? [Items order is randomized]

(1 Strongly support, 2 Somewhat support, 3 Somewhat oppose, 4 Strongly oppose)

*[Section2_Q6_a]* The production of news articles for mass media

*[Section2_Q6_b]* The production of scientific research manuscripts

*[Section2_Q6_c]* The analysis of large data sets

*[Section2_Q6_d]* The provision of public services (e.g., policing, theft-prevention, weather forecasting, public transit, etc.)

*[Section2_Q6_e]* The improvement of quality control in manufacturing processes or laboratory experiments

*[Section2_Q6_f]* The production of creative works (e.g., art, music, poetry, etc.)

*[Section2_Q6_g]* The provision of mental health therapy, in place of individual counseling

*[Section2_Q6_h]* The provision of personalized medical care for individuals (e.g., amount of pain medications to prescribe, screening of medical images, etc.)

**Section 3: Your Academic Experiences with Generative AI**

This section aims to gain insights into the professional views and attitudes of academic scientists toward AI use in academia.

**Please answer to the best of your knowledge and consider your academic experience with generative AI.**

*[Section3_Q7]* Have you personally ever used any generative AI systems (e.g., ChatGPT, Bard, Stable Diffusion, Midjourney, DALL-E)?

1 Yes

2 No

*Pre-skip logic*: if *[Section3_Q7]* = 2 (No), then skip *[Section3_Q8], [Section3_Q9], [Section3_Q10_ContinueToUse], [Section3_Q11], [Section3_Q12], [Section3_Q13_ContinueToUse]*

*[Section3_Q8]* Have you ever used generative AI for any of the following teaching activities? Please select all that apply.

1 Developing pedagogical materials

2 Integration in exams

3 Class exercise and experiment activity in using generative AI

4 Designing student assignments

5 Grading and evaluating student works

6 Mentoring students (e.g., email communication, recommendation letter, etc.)

7 Other (please specify): *[Section3_Q8_Other]*

8 I have never used generative AI for any teaching activity

*Pre-skip logic:* if *[Section3_Q8]* != 8, then show *[Section3_Q9]*

*[Section3_Q9]* How frequently do you use generative AI in your teaching activities?

1 Never

2 Rarely (less than once a month)

3 Occasionally (1-3 times a month)

4 Regularly (1-3 times a week)

5 Frequently (more than 3 times a week)

*Pre-skip logic:* if *[Section3_Q9]* != 1 (Never), then skip *[Section3_Q10]* but show *[Section3_Q10_ContinueToUse]*

*[Section3_Q10_ContinueToUse]* Do you plan to continue using generative AI in your teaching activities in the future?

1 Yes, definitely

2 Probably

3 No, I’m not planning to use it anymore

4 Unsure at the moment

*[Section3_Q10]* Do you plan to use generative AI in your teaching activities in the future?

1 Yes, definitely

2 Probably

3 No, I’m not planning to use it

4 Unsure at the moment

*[Section3_Q15]* Do you include any of the following policies regarding generative AI use in your course syllabi?

(1 Yes, 2 No)

*[Section3_Q15_a]* Allow use of generative AI with no restrictions.

*[Section3_Q15_b]* Allow use of generative AI, but ask students to engage critically with the tool.

*[Section3_Q15_c]* Allow use of generative AI, but ask students to cite it when used.

*[Section3_Q15_d]* Allow use of generative AI, but only for specific assignments.

*[Section3_Q15_e]* Prohibit use of generative AI.

*[Section3_Q15_f]* My course(s) does not lend itself to generative AI tools.

*[Section3_Q11]* Have you ever used generative AI for any of the following research activities? Please select all that apply.

1 Research conceptualization

2 Data curation

3 Data analysis

4 Funding acquisition (e.g., searching for potential funders)

5 Visualization

6 Writing - original draft

7 Writing - review and editing

8 Other (please specify): *[Section3_Q11_Other]*

9 I have never used generative AI for any research activity

*Pre-skip logic:* if *[Section3_Q11]* != 9, then show *[Section3_Q12]*

*[Section3_Q12]* How frequently do you use generative AI in your research activities?

1 Never

2 Rarely (less than once a month)

3 Occasionally (1-3 times a month)

4 Regularly (1-3 times a week)

5 Frequently (more than 3 times a week)

*Pre-skip logic:* if *[Section3_Q12]* != 1, then skip *[Section3_Q13]* but show *[Section3_Q13_ContinueToUse]*

*[Section3_Q13_ContinueToUse]* Do you plan to continue using generative AI in your research activities in the future?

1 Yes, definitely

2 Probably

3 No, I’m not planning to use it

4 Unsure at the moment

*[Section3_Q13]* Do you plan to use generative AI in your research activities in the future?

1 Yes, definitely

2 Probably

3 No, I’m not planning to use it

4 Unsure at the moment

*[Section3_Q16]* How confident are you of your ability to________? [Items order is randomized.]

(1 Not confident at all, 2 Not very confident, 3 Somewhat confident, 4 Very confident, 5 Extremely confident)

*[Section3_Q16_a]* Keep up with advances in generative AI technology

*[Section3_Q16_b]* Fully embrace generative AI technology in your teaching activities

*[Section3_Q16_c]* Fully embrace generative AI technology in your research activities

*[Section3_Q16_d]* Monitor the use of generative AI technology by your students

*[Section3_Q16_e]* Monitor the use of generative AI technology when reviewing grant applications

*[Section3_Q16_f]* Monitor the use of generative AI technology when reading the professional literature

*[Section3_Q16_g]* Monitor the use of generative AI technology when reviewing student admission applications

Section 4: University Policies

**Thank you so much for your participation. This last section asks about specific AI policies or guidelines in academia and in your institution.**

*[Section4_Q17]* In your opinion, which of the following, if any, should have primary responsibility for regulating generative AI in academic settings?

(1 Yes, 2 No)

*[Section4_Q17_a]* National professional associations (e.g., the Association of American Universities, the American Council on Education, etc.)

*[Section4_Q17_b]* Publishers

*[Section4_Q17_c]* Academic institutions

*[Section4_Q17_d]* The federal government

*[Section4_Q17_e]* Journal editors

*[Section4_Q17_f]* Supranational organizations (e.g., the United Nations, NAFSA: Association of International Educators, etc.)

*[Section4_Q18]* Are there any steps or guidelines regarding the responsible use of ChatGPT or similar generative AI language models now in place in your:

(1 Yes, 2 No, 3 Don't know)

*[Section4_Q18_a]* Department?

*[Section4_Q18_b]* College?

*[Section4_Q18_c]* University?

*Pre-skip logic*: if *[Section4_Q18_a]* =1 then show *[Section4_Q19], [Section4_Q20]*, otherwise, skip *[Section4_Q19], [Section4_Q20]*.

*[Section4_Q19]* How do your department’s guidelines restrict faculty or student use of generative AI in any way (e.g., teaching, class work, or research)?

1 Guidelines place restrictions only on Faculty use of generative AI.

2 Guidelines place restrictions only on Student use of generative AI.

3 Guidelines place restrictions on both Faculty and Student use of generative AI.

*[Section4_Q20]* Please briefly describe the restrictions pertaining specifically to using AI that your department places on faculty and/or students. [Open-end]

*[Section4_Q21]* Have you received any training or guidance on the ethical considerations associated with using generative AI in an educational or research context offered by your university?

1 Yes, I have received training/guidance.

2 No, I have not received training/guidance.

3 Unsure.

Thank you so much for participating in this survey!
