# Supplementary material for: Generative AI and academic scientists in US universities: Perception, experience, and adoption intentions
Source: PLoS One. 2025 Aug 28;20(8):e0330416. doi: 10.1371/journal.pone.0330416 (PMC12393709; doi:10.1371/journal.pone.0330416)
Supplement: Attachment — Submitted filename: Response_to_reviewers.pdf [file pone.0330416.s006.pdf]

Generative AI and Academic Scientists in US universities: Perception, Experience, and Adoption Intentions

(PONE-D-24-54545)

Comments from Review Editor: Thank you for submitting your manuscript to PLOS ONE. After careful consideration, we feel that it has merit but does not fully meet PLOS ONE's publication criteria as it currently stands. Therefore, we invite you to submit a revised version of the manuscript that addresses the points raised during the review process.

Authors' response:
We sincerely appreciate the opportunity to submit a revised version of our manuscript. We have carefully addressed the constructive feedback and suggestions provided by the two reviewers, and this document outlines the changes made to the manuscript in response to each of their points.

We trust that the revisions we have implemented align with your expectations and those of the reviewers.

Thank you for your time and consideration.

Yours sincerely,
The Authors

General Comments

The changes are marked in the document with track changes.
Summary of changes:

- New discussion section
- Substantial changes in the literature review
- Extended methodological details
- +10 new references
- +1000 new words
- Questionnaire available in the appendix
- Changes in the abstract

Reviewer 1: Comment 1

The authors present an interesting manuscript exploring the attitudes and experiences of academic scientific researchers toward generative AI. The article provides insightful and valuable contributions to this emerging area of study. However, many aspects require further clarification and refinement to enhance the manuscript's overall readability and impact.

Authors' response:
Thank you for your thoughtful feedback and appreciation of our work. We have carefully addressed your suggestions to improve clarity, readability, and overall impact. We appreciate your insights and look forward to your further comments.

Reviewer 1: Comment 2

*[**Abstract**]*

- *How many respondents has the study included?*
- *Considering the results you wrote in your abstract, what do you mean by the term cautious?*
- *STEM participants only: how did this influence your results?*

Authors' response:

- Thank you for highlighting the importance of specifying the number of respondents. We have clarified the abstract.
- By "cautious", we intended to reflect that academic scientists, while optimistic, remain aware of AI's potential risks such as misinformation and plagiarism.
- Focusing exclusively on STEM participants allowed for a concentrated examination of disciplines most closely interacting with AI, reflecting specific patterns unique to their educational and research contexts.

New abstract:

The integration of generative Artificial Intelligence (AI) into academia has sparked significant interest and debate among academic scientists. This paper explores the early adoption and perceptions of US academic scientists regarding the use of generative AI in teaching and research activities. To do so, the analysis focuses exclusively on STEM fields due to their high exposure to rapid technological advancements. Drawing from a nationally representative survey of 232 respondents, we examine academic scientists' attitudes, experiences, and intentions regarding AI adoption. Results indicate that 65% of respondents have utilized generative AI in teaching or research activities, with 20% applying it in both areas. Among those currently using AI, 84% intend

to continue its application, signaling strong confidence in its benefits. AI is most frequently used in teaching to develop pedagogical materials (51%) and in research for writing, reviewing, and editing tasks (40%). Despite significant concerns about misinformation, with 78% of respondents indicating it as their top concern regarding AI, there is widespread recognition of AI's potential impact on society. Most academic scientists have already integrated AI into their academic activities, demonstrating cautious yet optimistic adoption due to perceived risks. Furthermore, there is strong support for academic-led regulation of AI, highlighting the need for responsible governance to maximize benefits while minimizing risks in educational and research settings.

Reviewer 1: Comment 3

*[**Introduction**] What is particularly evident in the **STEM fields**? Does the field of AI really cause the growing number of students in this field? (Line 60)*

Authors' response:
We appreciate the reviewer's question. The text suggests a correlation between AI advancements and increased enrollment in STEM fields, but it does not claim direct causation. The growing number of students in STEM is influenced by multiple factors, including technological advancements, economic incentives, and labor market demand. To clarify this, we refined the sentence.

Changes in the text (in red):

Building on this transformative impact, generative AI's significance is particularly evident in Science, Technology, Engineering, and Mathematics (STEM) fields. The importance of these fields is underscored by the growing number of students pursuing STEM disciplines, a trend influenced by various factors and reflective of a country's economic development [8].

Reviewer 1: Comment 4

*[**Introduction**] You did not define generative AI. Only that it can produce **human-like texts**. But generative AI is more than that, right?*

Authors' response:
Thank you for this observation. Indeed, generative AI encompasses more than just text generation; it also includes image, video, and audio generation, among other capabilities. We have provided a broader definition.

Changes in the text (in red):

> *Fast forward to late 2022, the release of ChatGPT (https://openai.com/blog/chatgpt) marked a significant milestone in AI, democratizing access to advanced generative AI technologies. Generative AI refers to a subset of artificial intelligence that is designed to create new content, such as text, images, audio, video, and even code, based on patterns learned from large datasets [2]. Unlike traditional AI, which focuses on analysis and prediction, generative AI produces novel outputs that mimic human creativity and problem-solving. The rise of generative AI, particularly through chatbots powered by including Large Language Models (LLMs), further exemplifies the impact of these technologies on various domains.*

Reviewer 1: Comment 5

*[**Introduction**] This article explores the **early adoption** (Line 74). Why do you specifically mention the term early adoption, and not just adoption?*

Authors' response:

We appreciate this question. The term early adoption is used because the study focuses on the initial phase of generative AI integration among academic scientists and our dataset captures adoption within a limited time frame shortly after the launch of ChatGPT in November 2022. Since these technologies have only recently become widely accessible, it is crucial to distinguish between their initial uptake and more mature adoption stages. However, we added a clarification.

Changes in the text (in red):

> *Given the rapid global adoption of generative AI, this article explores the early adoption and perceptions of US academic scientists on the use of this technology in teaching and research activities. According to the innovation diffusion theory [13], early adopters are different from late adopters as they often face higher uncertainty and different motivations compared to later adopters. In this context, early adoption refers to the initial phase of AI integration before it becomes widespread in academia. While the study is centered on the United States, the insights can be considered reflective of broader international trends due to the influential role of US academia on a global scale [14].*

Reviewer 1: Comment 6

*[**Introduction**] In section 4… (line 95): does this section contain the results of the scoping review and the survey?*

Authors' response:
Thank you for pointing this out. Section 4 presents the survey findings, while the literature review synthesizes prior research. To avoid confusion, we clarified this in the text.

Changes in the text:

> *Section 'Methodology' describes the methods and data collection process. Section 'Results' presents the findings of the survey, detailing academic scientists' perceptions, experiences, and intentions regarding generative AI in teaching and research. Finally, section 'Conclusions' draws conclusions, synthesizing key insights and pointing out limitations of the study.*

Reviewer 1: Comment 7

*[**Literature review**] Could you clarify how the literature review was conducted? Specifically, what search strings did you use, and what were the criteria for determining an article's eligibility for inclusion?*

Authors' response:
Thank you for your comment. This literature review is part of the introduction and serves an introductory purpose rather than functioning as a separate research component. Our goal is to provide context for the survey by outlining key themes relevant to the study, not to conduct a systematic review, which requires a predefined methodology for article selection. To ensure clarity, we have repositioned this section as a subsection of the Introduction, making it explicit that the research component of the paper is solely based on the survey.

Additionally, our approach to the literature review was collaborative and interdisciplinary. Members of our research team from diverse academic backgrounds contributed to identifying relevant sources, combining traditional academic database searches with reference exploration within existing literature. This allowed us to provide a broad and representative overview of the academic discourse on generative AI in higher education and research.

Reviewer 1: Comment 8

*[**Literature review**] References 14 and 17 should be placed at the end of their respective sentences for clarity and alignment with academic citation standards.*

Authors' response:
Thank you for this observation. We adjusted the placement of these citations.

Changes in the text:

> *In its early applications in this context, AI was employed to streamline complex administrative tasks, enhancing efficiency in areas like admissions processes and predicting student retention rates, which are crucial for institutional planning [16,17]. Additionally, academic libraries have utilized AI to optimize their services to enhance accessibility and efficiency [18]. For educational practices, AI has also contributed to personalized learning through adaptive tutoring systems and has helped identify and address student learning obstacles [19,20], thus potentially improving educational outcomes.*

Reviewer 1: Comment 9

*[**Literature review**] In the statement, "Academic libraries have utilised AI to optimise their services to enhance accessibility and efficiency" (Line 106), could you elaborate on what specific services you are referring to?*

Authors' response:
Thank you for your question. AI has been integrated into academic libraries to optimize cataloging, improve search and discovery through intelligent recommendation systems, and automate reference services via AI-powered chatbots. Additionally, AI facilitates text and data mining, supports information literacy instruction through adaptive learning systems, and streamlines circulation with intelligent agent technologies. To provide greater clarity, we have revised the sentence accordingly.

Changes in the text:

> *In its early applications in this context, AI was employed to streamline complex administrative tasks, enhancing efficiency in areas like admissions processes and predicting student retention rates, which are crucial for institutional planning [16, 17]. Additionally, academic libraries have leveraged AI for automated cataloging, intelligent search tools, AI-driven reference services, and adaptive learning systems, improving accessibility and operational efficiency [18].*

Reviewer 1: Comment 10

*[**Literature review**] Why is automated grading categorized as generative AI rather than simply AI? (Line 119)*

Authors' response:
Thank you for your question. Automated grading is categorized as generative AI because certain advanced systems do more than just assess predefined answers—they generate evaluative feedback, paraphrase responses, and, in some cases, even provide model answers or restructured explanations. Unlike traditional AI used for multiple-choice grading, generative AI-based grading systems engage in text generation by evaluating open-ended responses, identifying patterns, and producing human-like explanations, which aligns with the defining characteristics of generative AI. We have clarified the sentence.

Changes in the text:

> *In higher education, generative AI supports a range of advancements, including personalized learning experiences, providing advanced research support, and enabling automated grading that involves text generation [23,24].*

Reviewer 1: Comment 11

*[**Literature review**] You wrote: "AI's potential to undermine creativity, hinder academic integrity, and exacerbate existing inequalities." How does AI contribute to exacerbating inequalities? Providing examples or further explanation would clarify this point.*

Authors' response:
We appreciate your request for clarification. AI can contribute to inequalities in higher education by reinforcing biases present in datasets and algorithms, which may disadvantage certain groups in educational and administrative processes. Additionally, the overreliance on AI in teaching, assessment, and decision-making can disproportionately affect those with limited access to these technologies, creating disparities in learning opportunities. The lack of transparency in AI's decision-making further complicates accountability, potentially leading to outcomes that disproportionately impact marginalized groups. To provide more clarity, we revised the sentence.

Changes in the text:

> *Notable issues include the risk of overreliance on AI affecting critical thinking [23,38] and AI's potential to undermine creativity, hinder academic integrity, and reinforce existing inequalities due to biases in datasets, algorithmic opacity, and disparities in access to AI-driven tools [39].*

Reviewer 1: Comment 12

[***Literature review***] *A reference is missing after the statement: "The unique capabilities and widespread adoption of generative AI have broadened and intensified these concerns."*

Authors' response:
Thank you for catching this omission. We added the appropriate reference to support this statement.

New reference:

> *Gruenhagen JH, Sinclair PM, Carroll J-A, Baker PRA, Wilson A, Demant D. The rapid rise of generative AI and its implications for academic integrity: Students' perceptions and use of chatbots for assistance with assessments. Comput Educ Artif Intell. 2024;7: 100273. doi:10.1016/j.caeai.2024.100273*

Reviewer 1: Comment 13

[***Literature review***] *In the third paragraph of the literature review, there appears to be inconsistent use of the terms "AI" and "generative AI." Could you clarify whether these terms are being used interchangeably or if there is a specific reason for the distinction?*

Authors' response:
Thank you for your observation. In the third paragraph of the literature review, we first discuss broader AI applications in higher education before narrowing the focus to generative AI. This progression is intentional, as it allows us to contextualize how AI has been used in general academic settings before addressing the specific challenges and opportunities posed by generative AI following their emergence.

Reviewer 1: Comment 14

*[**Literature review**] You wrote: "... is balanced by concerns over risks such as misinformation, plagiarism, and falsified research. For example, while generative AI can produce high-quality text suitable for publication, it has limited capacity for research design and data analysis" (Line 159). How does this example relate to the concerns mentioned, such as misinformation, plagiarism, and falsified research?*

Authors' response:
Thank you for pointing this out. The example highlights how generative AI's ability to produce high-quality text can contribute to misinformation and falsified research if the generated content appears credible but lacks proper research design and data analysis. Without rigorous methodological grounding, AI-generated academic texts risk introducing errors, fabricating data, or producing misleading conclusions. We revised the sentence to explicitly link this limitation to the stated concerns.

Changes in the text (in red):

> *For example, while generative AI can produce high-quality text suitable for publication, its limited capacity for research design and data analysis increases the risk of misinformation, fabricated findings, and academic integrity violations [54].*

Reviewer 1: Comment 15

*[**Literature review**] What does "the previous review" mean in Line 168?*

Authors' response:
Thank you for noting this ambiguity. "The previous review" refers to the previous paragraphs in the literature review section where we analyze existing research on AI and generative AI in higher education. To avoid confusion, we revise the wording.

Changes in the text (in red):

> *Understanding academic scientists' perceptions of generative AI is crucial because of its potential to radically transform the research and teaching landscapes in higher education, affecting vast segments of the US and international academic communities.*

Reviewer 1: Comment 16

*[**Literature review**] In "... benefits and challenges of generative AI" (Line 168), if you are referring to the previous paragraph, it seems the text pertains to AI in general rather than generative AI specifically.*

Authors' response:
Thank you for your observation. Following the recommendations for comment 15, we reviewed the sentence to ensure consistency in terminology.

Reviewer 1: Comment 17

*[**Literature review**] You wrote: "These include plagiarism, misinformation, and misidentification of AI-produced content as human work." and "For instance, in medical education, while there is optimism about AI's potential to reduce administrative burdens and enhance diagnostic accuracy, concerns persist about data privacy, the loss of personal contact with patients, and the adequacy of current AI technologies remain" (Lines 174-181). While your example in medical education provides meaningful insights, it does not directly align with the specific issues highlighted in the first sentence, such as plagiarism, misinformation, and the misidentification of AI-produced content. To ensure coherence, consider revising the example to better reflect these concerns or modifying the first sentence to encompass broader challenges associated with AI applications.*

Authors' response:
Thank you for your careful reading. We acknowledge that the example in medical education does not directly address plagiarism, misinformation, or the misidentification of AI-generated content. However, we included it as an illustration of how AI-related concerns manifest in highly specialized and complex domains. In this case, the risks involve overreliance on AI-generated medical content without human oversight and challenges in ensuring the accuracy and reliability of AI-assisted educational tools. To ensure coherence, we have revised the passage to clarify this connection while maintaining the relevance of the example.

Changes in the text (in red):

> *These risks affect various fields differently. For instance, in medical education, while there is optimism about AI's potential to reduce administrative burdens and enhance diagnostic accuracy, concerns persist about data privacy, the potential for AI-generated medical content to be overly relied upon without human oversight, and the challenges of ensuring the accuracy and reliability of AI-assisted educational tools [70].*

Reviewer 1: Comment 18

*[**Literature review**] Why do you not focus on generative AI rather then AI in general in line 8 (Line 198-208), as your research is also only focused on generative AI.*

Authors' response:

Thank you for your insightful comment. You are absolutely right that the focus should remain on generative AI, as it aligns with the scope of our research. We have revised the paragraph to ensure that it explicitly refers to generative AI rather than AI in general, maintaining consistency throughout the manuscript.

Reviewer 1: Comment 19

*[**Literature review**] I think the following sentence: To bridge this gap (line 214), should be subsentence. Material and methods*

Authors' response:

Thank you for your suggestion. We agree that restructuring this sentence as a subordinate clause would improve readability and coherence. We have revised it accordingly.

Changes in the text (in red):

> *However, few studies have jointly addressed STEM academic scientists' perceptions, experiences, and generative AI adoption intentions in both education and research. In order to bridge this gap, this study develops a comprehensive approach to better delineate the current state and more accurately understand the adoption intentions of academic scientists, which will be essential for tailoring educational methodologies, enhancing research practices, and designing regulation addressing concerns regarding the regulation of generative AI.*

Reviewer 1: Comment 20

*[**Material and methods**] Most important: the study can not be repeated. The reader has no idea what the survey entailed. Info is missing. Provide the exact questions and corresponding answer options that were explicitly asked during the survey for reproducibility.*

Authors' response:
Thank you for highlighting this issue. The complete set of survey questions and corresponding answer options are included in the AI survey dataset, which we have already submitted to the Roper Center data archive at Cornell University. The dataset is currently being processed. Once approved, researchers will be able to access the dataset, methodology, and survey instrument through the Roper Center.

Changes in the text (in red):

> *The questions underwent multiple rounds of review, revision, and internal pretesting by 10 SciOPS research team members. Through this iterative process of refinement, our survey instrument is reliable and have both internal and external validity. The full questionnaire is available in appendix B.*

Reviewer 1: Comment 21

*[**Material and methods**] When mentioning SciOPS for the first time, write out its full name and provide a brief explanation of what it is, instead of just using the abbreviation. This helps readers unfamiliar with the term understand its relevance.*

Authors' response:
Thank you for your suggestion. We revised the first mention of SciOPS.

Changes in the text (in red):

> *We make use of a unique database consisting of two sets of survey data from SciOPS (Scientist Opinion Panel Survey): an intake survey administered in the Spring of 2022 and 2023 to collect general socio-demographic data regarding SciOPS panelists, and another survey of panelists' attitudes towards AI that was administered in late 2023 (hereafter, the AI survey).*

Reviewer 1: Comment 22

*[**Material and methods**] The reference style for https://www.sci-ops.org/ is incorrect. Ensure it follows the required citation format for the journal or conference submission guidelines.*

Authors' response:
Thank you for your suggestion. We have included the website as a reference.

Changes in the text (in red):

> *SciOPS is a platform to improve science communication between university academic scientists and the public by surveying scientist opinions on current topics relevant to the science community and to society. Detailed information regarding SciOPS and past surveys administrated by this platform can be found at SciOPS website [75].*

Reviewer 1: Comment 23

*[**Material and methods**] The sentence on line 232 repeats the list of STEM fields: "six fields of STEM academic scientists biology, chemistry, civil and environmental engineering, computer and information science engineering, geography, and public health." Simplify it or rephrase it to avoid redundancy.*

Authors' response:
Thank you for your comment. Upon review, we see that the sentence does introduce the six STEM fields clearly, but we recognize that the wording could be streamlined for improved readability. We have revised the sentence to maintain clarity while avoiding potential redundancy.

Changes in the text (in red):

> *SciOPS panel members were recruited from a full sample frame that includes around 18,500 randomly selected PhD-level academic scientists employed by Carnegie-designated Research Extensive and Intensive (R1) universities across the US, representing six STEM fields: biology, chemistry, civil and environmental engineering, computer and information science engineering, geography, and public health. The SciOPS research team used probability sampling to randomly select R1 universities and collected the name and contact information of the tenure and tenure track faculty (i.e., assistant, associate, and full professors), and non-tenure track researchers with PhDs from each sampled department website. Table A3 in appendix shows the number of randomly selected institutions by fields.*

Reviewer 1: Comment 24

*[**Material and methods**] The term "AAPOR" should be fully spelt out and briefly explained when first mentioned. For example, "AAPOR (American Association for Public Opinion Research)" if that is the intended meaning.*

Authors' response:
Thank you for your suggestion. We now ensure that AAPOR is spelled out in full the first time it appears in the text, along with a brief explanation of its relevance to the study.

Changes in the text (in red):

> *Of these, a total of 1,365 eligible academic scientists consented to become SciOPS panel members, with a recruitment rate of 7.5%, calculated according to the guidance of American Association for Public Opinion Research (AAPOR) Recruitment Rate 6 (RECR RR6), which estimates the proportion of successfully recruited participants by assuming no unknown cases are eligible and including partial responses [76].*

Reviewer 1: Comment 25

*[**Material and methods**] The use of "etc." on line 239 is vague. Specify what additional items or categories it includes to provide clarity.*

Authors' response:
Thank you for your suggestion. We acknowledge that "etc." can be vague in an academic context. To improve clarity, we specified the additional socio-demographic variables included in the survey.

Changes in the text (in red):

> *The intake survey was dispensed to all 1,365 members to capture their general socio-demographic information, including birth year, self-reported gender, race, ethnicity, citizenship, academic rank, and academic discipline.*

Reviewer 1: Comment 26

*[**Material and methods**] These lines appear to be part of the results section. Move them to the appropriate section to maintain logical flow and structure.*

Authors' response:

Thank you for your observation. However, it is unclear which specific lines you are referring to as misplaced. Since this section describes the construction of the dataset and its characteristics, we believe it is more appropriate to keep all this information here rather than moving it to the results section

Reviewer 1: Comment 27

*[**Material and methods**] For clarity and compliance, include the Institutional Review Board (IRB) approval number or ID after the sentence: "Both surveys were approved by the Institutional Review Board at Arizona State University."*

Authors' response:

Thank you for your suggestion. We have included the IRB approval number or ID to ensure clarity and compliance with ethical research standards.

Changes in the text (in red):

> *Both surveys were approved by the Institutional Review Board at Arizona State University (IRB Approval No. STUDY00012476).*

Reviewer 1: Comment 28

*[**Material and methods**] The sentence starting with "The results indicate..." (lines 275–278) belongs in the results section rather than its current location. Relocate it to ensure consistency and proper organisation*

Authors' response:

Thank you for your observation. Since this sentence refers to an additional methodological analysis rather than presenting primary findings, we believe it is appropriate to keep it within this section. However, to improve the transition into the results section, we revised the wording to better connect the methodological discussion with the subsequent presentation of findings.

Changes in the text (in red):

To assess the representativeness of our sample, we also conducted non-response bias analysis (see appendix A for details). The analysis shows minimal differences between the completed sample and the initial sample frame of academic scientists, and no significant differences between our sample and the panel members invited to participate in the survey. These findings confirm that the dataset provides a reasonable representation of US-based academic scientists.

Reviewer 1: Comment 29

*[**Results**] All in all, there are several sentences that seem to refer to opinions/conclusions rather than on findings.*

Authors' response:

Thank you for your observation. We have carefully reviewed the results section to ensure that it presents empirical findings rather than interpretations or conclusions. Any statements that reflect opinions or broader discussions have been removed or reworded to align with the data presented.

Reviewer 1: Comment 30

*[**Results**] Ensure consistent use of "generative AI" and "AI" throughout the results section. Often, "AI" is used where "generative AI" would be more appropriate.*

Authors' response:

Thank you for your careful reading. We acknowledge that there may be inconsistencies in terminology. We have carefully reviewed the results section to ensure that "generative AI" is used whenever the discussion specifically pertains to AI models that generate content, while "AI" has been retained only when referring to broader artificial intelligence technologies.

Reviewer 1: Comment 31

*[**Results**] The results would benefit from an analysis of differences in attitudes and concerns regarding generative AI among academic scientists across various STEM disciplines. Could you explore and include this aspect in your findings?*

Authors' response:

Thank you very much for this valuable suggestion. We agree that exploring potential differences in attitudes and concerns regarding generative AI across various STEM disciplines is a relevant analytical direction. While a comprehensive disciplinary comparison is beyond the scope and design of the present study, and would require a more balanced sample across fields to be conclusive, we acknowledge that our data can offer preliminary insights in this regard. As a first step, and in response to your comment, we conducted a one-way ANOVA analysis on the weighted data to explore whether disciplinary background is associated with variation in key attitudes. Although the results do not allow for definitive conclusions, they do reflect significant differences ($p < 0.05$) in three areas: (1) concern about misinformation, and support for generative AI in (2) the production of news articles for mass media, and (3) the production of scientific research manuscripts. These findings, are now briefly reported in the manuscript to acknowledge possible disciplinary variation.

Changes in the text (in red):

> *This is juxtaposed with 11% who use generative AI exclusively for research and a smaller 9% who apply it solely to teaching.*
>
> *To assess potential disciplinary variation in attitudes toward generative AI, a one-way ANOVA was conducted across all survey items. Although some field subgroups are small, statistically significant differences ($p < 0.05$) were found only in a few items, namely concern about misinformation and support for the use of generative AI in the production of news articles for mass media and the production of scientific research manuscripts.*

Reviewer 1: Comment 32

*[**Results**] The sentence, "Building on the understanding that generative AI will significantly impact daily life in the United States" (line 300), is an assumption and should not appear in the results section. Replace it with a neutral, evidence-based statement or move it to the discussion section if it is necessary for interpretation.*

Authors' response:
Thank you for your observation. This revision has been made as part of the broader changes addressing Comment 29. We revised the results section to ensure it remains evidence-based and clearly attributed to survey respondents rather than stated as an assumption.

Changes in the text (in red):

> *Survey responses indicate that a majority of academic scientists (93%) believe that generative AI will have a significant impact on daily life in the United States (Fig 1). The potential effects of climate change also register as a major point of academic concern, albeit slightly less so than generative AI, indicating a sense of urgency regarding environmental shifts.*

Reviewer 1: Comment 33

*[**Results**] Define what qualifies someone as an "early adopter" of AI. Specify the criteria or timeframe, as the current mention in line 318 leaves this unclear.*

Authors' response:
Thank you for your suggestion. We acknowledge that the term "early adoption" needs further clarification. In this study, early adopters are defined as academic scientists who have integrated generative AI into their teaching or research despite the absence of widespread institutional policies or formalized best practices at the time of data collection (2023). This group demonstrates a proactive approach to AI use, engaging with the technology before it becomes a standardized component of academic work. We clarified the definition of "early adoption" within this paragraph.

Changes in the text (in red):

> *Specifically, 40% of surveyed academic scientists report using generative AI in teaching and research, demonstrating an early adoption—defined here as the integration of generative AI into academic activities at a stage when its use was still emerging and not yet widely formalized—despite the acknowledged risks. In contrast, 25% utilize generative AI for alternative functions not directly related to their primary roles, and 35% have not engaged with AI personally.*

Reviewer 1: Comment 34

*[**Results**] In the sentence, "20% of academic scientists report using AI in both teaching and research, illustrating a robust integration into the academic environment" (line 321), consider whether "robust" is the right term.*

Authors' response:
Thank you for your observation. This interpretation has been removed as part of the broader revisions made in response to Comment 29. We have ensured that the sentence now presents the data neutrally, without implying the extent of integration beyond what is directly supported by the survey findings.

Reviewer 1: Comment 35

*[**Results**] The statement, "Yet, there is a small segment, 20%, considering its future use, with 17% likely and 3% certainly planning to incorporate AI into their teaching," is unclear. Specify 17% and 3% of what. This follows a prior sentence mentioning 80% expressing reservations, which makes the proportions ambiguous. Rephrase for clarity and logical flow.*

Authors' response:
Thank you for your suggestion. We recognize that the percentages in this section may be ambiguous in relation to the preceding sentence. To improve clarity, we have explicitly stated that the 17% and 3% figures refer to the 20% of respondents who are considering future AI use in teaching and restructure the sentence for better logical flow.

Changes in the text (in red):

> *A majority, 80%, express reservations about adopting generative AI: within this group, 45% are inclined not to use it, and 35% remain undecided. Only 20% of current non-users express openness to future use; within this group, 85% (i.e., 17% of the total) state they are likely to adopt it, while 15% (i.e., 3% of the total) are certain they will incorporate it into their teaching.*

Reviewer 1: Comment 36

*[**Results**] You stated: "where 40% of users find value" (Line 358) and earlier wrote: "a substantial 40% are employing AI specifically within the domains of teaching and research" (Line 317). Is this referring to the same 40% of participants, or does the latter imply that 40% of the earlier 40% find value in AI? Please clarify this distinction for consistency.*

Authors' response:
Thank you for your careful reading. We acknowledge that the repeated reference to "40%" in different contexts may create confusion. To clarify, the first 40% refers to the proportion of all surveyed academic scientists who use generative AI in teaching and research, whereas the second 40% refers to the proportion of generative AI users who find value in specific research tasks such as writing, reviewing, and editing. To avoid ambiguity, we have refined the wording in both sections.

Changes in the text (in red):

> *A sizable 65% of academic scientists have personally used generative AI to some extent. Specifically, 40% of surveyed academic scientists report using generative AI in teaching and research, demonstrating an early adoption—defined here as the integration of generative AI into academic activities at a stage when its use was still emerging and not yet widely formalized—despite the acknowledged risks.*
>
> *Nearly half (46%) of researchers employ generative AI less than once a month, indicating a tentative exploration, and about a quarter (24%) use it on a weekly basis. Additionally, among academic scientists who only use generative AI in research, 40% report finding value in its applications for writing, reviewing, and editing. Significantly, generative AI's role in data analysis (32%) and especially in research conceptualization (28%) marks a pivotal shift, showcasing its transformative potential not just in routine tasks but in critical thinking and idea generation phases.*

Reviewer 1: Comment 37

*[**Results**] You wrote: "This distribution of responsibilities suggests a preference for an academic-led approach to AI regulation, complemented by governmental and international oversight, to effectively address the intricacies of AI's integration into academic settings." (Line 393-396). This is an insightful observation, but it remains abstract. Could you elaborate on what specific actions or frameworks academics,*

governments, or international bodies could implement to address these intricacies? Adding 1-2 sentences in the discussion section would strengthen your argument.

Authors' response:
Thank you for your valuable feedback. We appreciate your suggestion to provide more concrete examples of regulatory actions concerning AI in academia. To address this, we have expanded the discussion section to highlight how different regulatory stakeholders are responding to AI integration. Specifically, universities are developing institutional policies to govern AI use in education and research, while academic publishers and journal editors are establishing guidelines on AI-assisted writing and reviewing processes. Additionally, international organizations such as UNESCO have proposed global frameworks for AI governance in education and research. These evolving regulatory efforts underscore the need for a multi-layered governance approach that balances academic autonomy with national and international oversight. We have incorporated these references in the results to strengthen our argument.

Changes in the text (in red):

> *Although a majority (71%) of respondents support some federal involvement, their preferences clearly emphasize cautious, evidence-based policies rather than outright governmental bans. Experimental survey data explicitly indicate that academic scientists' regulatory positions vary significantly depending on how AI-related risks are presented, reflecting context-dependent rather than absolute regulatory stances. Current regulatory practices already align with these academic preferences. Universities are actively developing internal AI governance frameworks [52], academic publishers have begun establishing clear editorial standards for AI-generated content [81], and international organizations such as UNESCO have advanced global governance principles to shape national AI policies [82].*

Reviewer 1: Comment 38

*[**Results**] You wrote: "revealing that the way questions are framed significantly influences their responses." (Line 414) It seems the responses were not influenced by how questions were framed but rather by the way answers were provided. This distinction is important. Please clarify the statement accordingly.*

Authors' response:
Thank you for this important clarification. We have revised the statement to accurately reflect that the influence observed was due to how response options were structured, rather than the framing of the questions themselves.

Changes in the text (in red):

> *Participants were randomly assigned one of four variations of the question, covering aspects such as outright banning versus not banning generative AI technologies and the inclusion or exclusion of a middle response category. This methodological experiment indicates that the format in which response options were structured significantly influenced participants' answers.*

Reviewer 1: Comment 39

*[**Results**] In your methodology, you wrote: "SciOPS panel members were recruited from a full sample frame that includes around 18,500 randomly selected PhD-level academic scientists employed by Carnegie-designated Research Extensive and Intensive (R1) universities across the US with appointments in six fields of STEM academic scientists – biology, chemistry, civil and environmental engineering, computer and information science engineering, geography, and public health." However, in the results section (Line 440), you stated that findings are limited to biologists, biochemists, and civil & environmental engineers. Why were computer engineers and geography scientists excluded from the analysis? Please explain and ensure this is clarified in the text.*

Authors' response:

Thank you for catching this inconsistency. This was an unintended error in the results section. We have corrected it to reflect that our findings are based on responses from all six STEM disciplines included in the study, as described in the methodology.

Changes in the text (in red):

> *First, although based on probability sampling, the findings reflect responses from academic scientists across the six STEM disciplines included in the study, which may not represent the broader academic community*

Reviewer 1: Comment 40

*[**Results**] You mentioned: "while the survey presents data by gender, it did not inquire about race or ethnicity, preventing an assessment of differences across these dimensions." (Line 442). This is a valid limitation. However, did you also consider assessing variations across scientific professions within STEM disciplines? Since this is a study about generative AI in academia, understanding differences across various*

scientific fields could provide valuable insights. Please address this or justify its omission.

Authors' response:
Thank you for your thoughtful suggestion. As mentioned in our response to a related comment (31), the analysis of disciplinary differences in attitudes toward generative AI is being explored in a separate study currently in development. This follow-up research employs a more detailed statistical methodology and a dedicated analytical framework to rigorously assess whether significant variations exist across STEM disciplines. Given the comprehensive nature of our current manuscript, integrating such an in-depth statistical exploration here would significantly increase its length and complexity. For this reason, we have opted to address this aspect in a forthcoming publication that will provide a more focused discussion on disciplinary variations.

Reviewer 1: Comment 41

*[**Results**] You used the term "AI-generated content" (Line 450). Is this terminology distinct from "Generative AI"? If so, please clarify the distinction in the text to ensure consistency and precision in the use of terminology.*

Authors' response:
Thank you for your comment, "generative AI" refers to the technology itself, while "AI-generated content" describes the output it produces. This distinction is intentional, and we have ensured consistency in the text where appropriate.

Reviewer 1: Comment 42

*[**Conclusions**] R.485: by whom should this be done?*

Authors' response:
Thank you for your comment. However, as part as Comment 43, we have removed this section and instead incorporated an expanded discussion section. This new section provides a more in-depth analysis of key findings, contextualizing them within the broader academic and policy landscape.

Reviewer 1: Comment 43

*[**Conclusions**] A real in depth discussion is missing.*

Authors' response:
Thank you for your comment. We have removed the conclusions section and replaced it with a comprehensive discussion of over 1,000 words. This new section provides a detailed analysis of our findings, situating them within relevant academic and policy contexts.

Reviewer 1: Comment 44

*[**NB**] The data are 'available'. Is this made anonymous? How is this done?*

Authors' response:
Thank you for your question. The data used in this study have been fully anonymized to protect respondent privacy, with no personally identifiable information collected or stored. The dataset has already been submitted to the Roper Center and is currently under review for public release. It 24ill be made publicly available upon approval.

Reviewer 2: Comment 1

The manuscript presents an insightful study on the integration of Generative AI in academia, focusing on US academic scientists' adoption, perceptions, and regulatory concerns. The topic is highly relevant, and the study provides valuable data from a nationally representative survey of STEM faculty. The findings contribute to ongoing discussions on AI's role in education and research. However, certain areas require further clarification and expansion, particularly regarding methodology, theoretical grounding, and the discussion of ethical concerns.

Authors' response:
Thank you for your thoughtful feedback and recognition of our study's relevance. We have carefully addressed your suggestions, enhancing the methodology, theoretical grounding, and discussion of ethical concerns.

Reviewer 2: Comment 2

The manuscript would benefit from engagement with established technology adoption models, such as the Technology Acceptance Model (TAM) or Unified Theory of Acceptance and Use of Technology (UTAUT).

Integrating these frameworks would help contextualize the faculty's perceptions and concerns regarding AI adoption.

Authors' response:
Thank you for your valuable suggestion. We have carefully revised the literature review to incorporate key frameworks, including the Technology Acceptance Model (TAM) and the Unified Theory of Acceptance and Use of Technology (UTAUT), alongside other relevant theories such as the Theory of Reasoned Action (TRA), the Theory of Planned Behavior (TPB), and the Innovation Diffusion Theory.

In our updated literature review section, we highlight how these models help explain academic scientists' perceptions of generative AI, emphasizing the role of personal beliefs, attitudes, confidence, readiness, and past experiences in shaping technology acceptance. This addition strengthens the contextualization of faculty concerns and adoption behaviors while reinforcing the study's foundation in well-established theoretical frameworks.

Changes in the text (in red):

> *Understanding academic scientists' perceptions of generative AI is crucial because of its potential to radically transform the research and teaching landscapes in higher education, affecting vast segments of the US and international academic communities. Theories such as the Theory of Reasoned Action (TRA) [58], the Theory of Planned Behavior (TPB) [59], the Technology Acceptance Model (TAM) [60], the Innovation Diffusion Theory [61] and the Unified Theory of Acceptance and Use of Technology (UTAUT) [62] provide valuable frameworks for examining technology acceptance. Cumulatively, they highlighted the importance of personal beliefs and attitudes, including concerns [63] confidence and readiness [60,64], as well as past experiences [64] in shaping technology acceptance. However, the implications of AI extend beyond adoption frameworks; AI's integration into academic environments presents unprecedented opportunities and significant uncertainties, making it imperative to closely monitor how these changes unfold over time.*

Reviewer 2: Comment 3

While the study is based on a nationally representative survey, key methodological details are missing, including:
- Sampling technique and representativeness of different institution types.
- Survey response rate and potential response bias.
- Validation of survey questions to ensure reliability and relevance.

Providing more transparency in these areas would enhance the study's rigor.

Authors' response:
Thank you for your comments on our sampling and survey design.

- We employed a two-stage sampling design in this study. All SciOPS panel members were recruited from the same type of institution– Carnegie-designated Research Extensive and Intensive (R1) universities in the US. We further clarify our sampling strategies in our manuscript.
- The survey response rate is 29.9%. We reported it in line 310 to 313. We have also conducted a non-response bias analysis and attached detailed results in Appendix A. Briefly, there were no significant demographic differences by gender, academic field, or academic scientists rank between respondents and the sample invited for this survey.
- To validate the survey questions, the SciOPS research team designed them based on literature on technology adoption among academic scientists. The questions underwent multiple rounds of review and revision by 10 SciOPS research team members. After programming the survey into the survey software, the entire SciOPS team further conducted three rounds of internal pretesting and revision. Additionally, we allowed respondents to email concerns and comments about the survey after they completed it. Through this iterative process of refinement, we ensured that our survey instrument was reliable and has both internal and external validity. We briefly mention our validation process in the method section.

Changes in the text:

Detailed information regarding SciOPS and past surveys administrated by this platform can be found at SciOPS website [75]. We employed a two-stage sampling design in this study. At the first stage, SciOPS panel members were recruited from a full sample frame that includes around 18,500 PhD-level academic scientists employed by Carnegie-designated Research Extensive and Intensive (R1) universities across the US, representing six STEM fields: biology, chemistry, civil and environmental engineering, computer and information science engineering, geography, and public health. The SciOPS research team used probability sampling to randomly select R1 universities and collected the name and contact information of the tenure and tenure track faculty (i.e., assistant, associate, and full professors), and non-tenure track

researchers with PhDs from each sampled department website. Table A3 in Appendix A shows the number of randomly selected institutions by field.

Table A3. Number of randomly selected institutions for sampling scientists

Fields	Number of randomly selected R1 institutions	Number of all R1 institutions
Biology	106	131
Civil and environmental engineering	46	131
Geography	46	131
Public health	61	61
Computer and information science engineering	125	131
Chemistry	62	131

A total of 1,365 eligible academic scientists consented to become SciOPS panel members, with a recruitment rate of 7.5%, calculated according to the guidance of American Association for Public Opinion Research (AAPOR) Recruitment Rate 6 (RECR RR6), which estimates the proportion of successfully recruited participants by assuming no unknown cases are eligible and including partial responses [76]. The intake survey was dispensed to all 1,365 members to capture their general socio-demographic information, including birth year, self-reported gender, race, ethnicity, citizenship, academic rank, and academic discipline.

In the second stage, 777 SciOPS panel members were randomly selected from all members and invited to participate in this survey. To construct valid survey questions, the SciOPS research team designed them based on literature on technology adoption among academic scientists. The questions underwent multiple rounds of review, revision, and internal pretesting by 10 SciOPS research team members. Through this iterative process of refinement, our survey instrument is reliable and have both internal and external validity. The full questionnaire is available in appendix B.

Reviewer 2: Comment 4

While the study mentions misinformation concerns (78%), it could also discuss other ethical implications, such as:
- Bias in AI models and how it affects academic use.
- Data privacy issues in AI-generated content.
- Plagiarism detection challenges in AI-assisted research.

A deeper engagement with these ethical dimensions would strengthen the manuscript's contribution.

Authors' response:
Thank you for your valuable feedback. In response to your suggestion regarding additional ethical implications, we have incorporated a dedicated discussion section. One of the paragraphs specifically addresses bias in AI models, data privacy concerns, and plagiarism detection challenges in AI-assisted research.

Changes in the text (in red):

> *While the study emphasizes misinformation concerns, other ethical implications warrant deeper exploration. The inherent bias in generative AI models, stemming from biased training data, significantly influences academic use by potentially perpetuating discriminatory outcomes, thus affecting fairness and equality within educational contexts [39]. Data privacy concerns also merit critical attention, especially regarding generative AI's role in processing and sharing sensitive academic and research data. For instance, AI-driven qualitative analysis methods raise important questions about ethical standards for data management [57]. Additionally, plagiarism detection presents notable challenges due to generative AI's capacity for creating highly sophisticated and indistinguishable academic content [80]. The resultant inability to reliably identify AI-generated content complicates existing plagiarism detection methods, demanding the development of more sophisticated identification systems or substantial revisions to current academic integrity standards.*

Reviewer 2: Comment 5

The study focuses on STEM faculty, which may limit generalizability to the humanities, social sciences, and international academic institutions.
Expanding the discussion on how AI adoption may differ across disciplines would enhance the broader relevance of the findings.

Authors' response:
Thank you for your valuable feedback. In response to your comment regarding the generalizability of our study beyond STEM disciplines, we have created a dedicated discussion section addressing this issue that explicitly acknowledges the study's limitations in focusing on STEM faculty.

Reviewer 2: Comment 6

While the study states that faculty support academic-led AI regulation, it does not provide concrete policy recommendations or engagement with existing AI governance frameworks.

Authors' response:

We appreciate your thoughtful feedback. We have incorporated this discussion into our manuscript. Specifically, we highlight how faculty regulatory positions are context-dependent rather than absolute, and we reference current regulatory practices that align with academic perspectives. Additionally, we discuss ongoing developments in AI governance, including university-led initiatives, editorial standards by academic publishers, and international frameworks such as those proposed by UNESCO.

Changes in the text (in red):

> *Although a majority (71%) of respondents support some federal involvement, their preferences clearly emphasize cautious, evidence-based policies rather than outright governmental bans. Experimental survey data explicitly indicate that academic scientists' regulatory positions vary significantly depending on how AI-related risks are presented, reflecting context-dependent rather than absolute regulatory stances. Current regulatory practices already align with these academic preferences. Universities are actively developing internal AI governance frameworks [52], academic publishers have begun establishing clear editorial standards for AI-generated content [82], and international organizations such as UNESCO have advanced global governance principles to shape national AI policies [82].*

Reviewer 2: Comment 7

Expanding this discussion with references to current AI policies in education would improve the impact of the study's findings.

Authors' response:

Thank you for your insightful feedback. We fully agree with your observation regarding the need to expand the discussion on current AI policies in education. In response, we have created a comprehensive discussion section of over 1,000 words, incorporating references to relevant studies and highlighting key differences and similarities in existing AI policies.
