# Supplementary material for: Generative AI and academic scientists in US universities: Perception, experience, and adoption intentions
Source: PLoS One. 2025 Aug 28;20(8):e0330416. doi: 10.1371/journal.pone.0330416 (PMC12393709; doi:10.1371/journal.pone.0330416)
Supplement: Attachment — Submitted filename: Response_to_reviewers_auresp_2.pdf [file pone.0330416.s007.pdf]

Generative AI and Academic Scientists in US universities: Perception, Experience, and Adoption Intentions
(PONE-D-24-54545)

<u>Comments from Review Editor</u>: Thank you for submitting your manuscript to PLOS ONE. After careful consideration, we feel that it has merit but does not fully meet PLOS ONE's publication criteria as it currently stands. Therefore, we invite you to submit a revised version of the manuscript that addresses the points raised during the review process.

The reviewers has recommended further changes and improvement on the manuscript. Please see reviewers' comments.

Authors' response:
We sincerely appreciate the opportunity to submit a revised version of our manuscript. We have carefully addressed the constructive feedback and suggestions provided by the two reviewers, and this document outlines the changes made to the manuscript in response to each of their points.

We trust that the revisions we have implemented align with your expectations and those of the reviewers.

Thank you for your time and consideration.

Yours sincerely,
The Authors

Reviewer #3: Comment 1

"First, despite being based on probability sampling, the findings are restricted to biologists, biochemists, and civil & environmental engineers," states Line 440 on Page 19. names "biochemists" inaccurately because the study is about biology, not biochemistry. "Biologists, chemists, and civil & environmental engineers" is the correct phrase.

Authors' response:
Thank you to the reviewer. This paragraph was removed in response to other comments, and the inconsistency has been resolved. We have also reviewed the entire text to ensure that the same issue does not occur elsewhere.

Reviewer #3: Comment 2

Inconsistencies in Style:
Use of "AI" vs. "generative AI": The term "AI" and "generative AI" are used interchangeably throughout the manuscript, however the differences are not always made clear. To fit with the study's aim, the sentence "A sizable 65% of academic scientists have personally used AI" on page 14, line 317, for instance, may be changed to "generative AI." Early in the article, think about standardising vocabulary or defining "AI" as "generative AI."

Authors' response:
Thank you for your observation. The ambiguity in the use of 'AI' and 'generative AI' has been resolved throughout the manuscript. Additionally, at the very beginning of the manuscript, we have included a brief clarification differentiating both terms.

Changes in the text:

> *Generative AI refers to a subset of artificial intelligence that is designed to create new content, such as text, images, audio, video, and even code, based on patterns learned from large datasets [2]. Unlike traditional AI systems, which function based on pre-defined rules, generative AI systems demonstrate greater functional autonomy and ability to perform intelligent behaviors in a way that mimic human creativity by learning patterns and behaviors from data [3]. The rise of generative AI, particularly through chatbots powered by including Large Language Models (LLM), further exemplifies the impact of these technologies on various domains. The rapid development of chatbots beyond ChatGPT has sparked considerable interest [4].*

Reviewer #3: Comment 3

Overall Evaluation
The manuscript satisfies PLOS ONE's standards for clarity, accuracy, and lack of ambiguity since it is written in standard English and presented in an understandable manner. The material is understandable to the intended audience of academic scholars, the language is formal and precise, and the structure makes sense. The majority of the text is free of significant typographical or grammatical problems, and the few that are found are small and simple to fix when it is revised.

Authors' response:
Thank you very much to the reviewer for your kind words. We have reviewed the paper carefully to identify and correct all typo and grammatical errors.

Reviewer #4: Comment 1

The survey conducted here is on a very small sample, making this manuscript fewly relevant but this is compensated by a deep and intructing litterature review that led me to recommend the acceptance. Furthermore, the authors took a great caution in answering the concerns of the first round of reviewing, gicing more value to the manuscript.

Authors' response:
Thank you very much for your thoughtful review and kind recommendation. Following your suggestions and those of the other reviewers, we have continued to improve the manuscript, especially by making the literature review more structured and fluid. We truly appreciate your valuable feedback.

Reviewer #4: Comment 2

In answered the data were available but it is still subject to approval and not available throught the article now.

Authors' response:
The reviewer is right, this inconsistency is due to the fact that the complete set of survey questions and corresponding answer options (included in the AI survey dataset) was submitted to the Roper Center data archive at Cornell University. The dataset is currently being processed. However, we expect to receive the public link soon, as we informed the Roper Center that the manuscript is in an advanced stage of peer review, based on what the editors have communicated, and will share it either before the next review round or, if the manuscript is accepted, during the proof stage so it can be correctly included in the final version.

Reviewer #4: Comment 3

I would nevertheless make the following suggestions :
1. Line 33 : "Results indicate that 65% of scientists" I would rather say that "Results indicate that 65% of respondants".
2. Line 210 : I am not sure the "Chubb et al." is relevant.
3. Line 253 & 559 & 562 :Science told me there is only one human race, thus differencing race and ethnicity seems abusive to me.
4. Line 280 : "The margin of sampling error for the AI survey estimates is +/-6.4 percentage points". The authors should state that this is for a population of 18 500 and a 95% CI.
5. Line 317 : "The potential effects of climate change also register as a major point of academic concern, albeit slightly less so than generative AI, indicating a sense of urgency regarding environmental shifts." But these are within the margin of error.
6. Line 448 : "Participants were randomly assigned one of four variations of the question". It would seem interesting to provide the margin of error for these sub-groups to the readers.
7. Line 483 : Generative AI is predominantly used in educational settings for developing pedagogical materials (51%) and in research primarily for writing, reviewing, and editing tasks(40%)" but it only seems since when margin of errors are taken into account this can be untrue.

Authors' response:
Thank you to the reviewer.
1. Done.
2. We agree, this sentence is not fully aligned with the paragraph. Since the paragraph is also somewhat lengthy, we have removed it to improve clarity and readability.
3. We share this reviewer's opinion and have removed references to race/ethnicity from the manuscript.
4. Correct, this information has been included.
5. This warning has been included in both the results section and the discussion section, as part of the study's limitations.
6. The 95% confidence intervals for these subsamples are, of course, wider. Specifically, the confidence intervals for each subsample are: n=77, ci= +/-11.1%; n=52, ci= +/-13.6%; n=50, ci= +/-13.8%. This information has been added as a note to Figure 5.
7. The reviewer is correct; however, since we have already addressed this situation in the methodology, at the beginning of the results section, and in the discussion, we believe it is not necessary to include an explicit warning each time two values fall within the margin of error. Doing so repeatedly would interrupt the flow of the text and reduce its clarity.

Changes in the text:

1. Results indicate that 65% of respondents have utilized generative AI in teaching or research activities, with 20% applying it in both areas.
4. The margin of sampling error for the AI survey estimates is +/-6.4 percentage points, based on a population of 18,504 and a 95% confidence level.
5. *In results section*: The potential effects of climate change also register as a major point of academic concern, albeit slightly less so (88%) than generative AI (though

this difference falls within the margin of error), indicating a sense of urgency regarding environmental shifts

In discussion section: First, although based on probability sampling, the findings reflect responses from academic scientists across the six STEM disciplines included in the study, which may not represent the broader academic community. Additionally, as with any cross-sectional study, the findings reflect the period of data collection (late 2022–early 2023), capturing attitudes and experiences during the early adoption phase; perspectives may have since evolved. Smaller percentage differences should be understood within the survey's overall margin of error (±6.4%), though this does not affect the general interpretation of results, which point to trends in academic scientists' attitudes and experiences.

6. In Fig 5 caption: Fig 5. Methodological experiment results: (a) Current opinion about the role of the government in regulating generative AI; (b) Current opinion about the role of the government in regulating generative AI, considering the possible ethical and unintended consequences it may have. Confidence intervals and sample sizes for each subsample are as follows: (a1) n = 77, CI = ±11.1%; (a2) n = 50, CI = ±13.8%; and (b1 and b2) n = 52, CI = ±13.6%.

Reviewer #4: Comment 4

I could also discuss the following the limits of this article :
- Sample size: The small sample size leads to an important margin of errors but more importantly reduces the relavance or significance of sub-groups analysis.
- Not all STEM widely represented (Geographs and civil engineers representing 66% of the cohort). This can both influence the result and the value of the data presented here and should be discussse omre in depth, since geographs seem to be more early adopters in bibliography. The signifiance upon other fields might also be discussed (For example, Biology with 16 answers might have very different behaviours that would not be highlighted by the present studies. Bibliography might also help on this point).
- It would also be interesting exploring deeper the results depending on the status of the respondants, whether professors, associates, or doctorants.

Authors' response:
We appreciate the reviewer's thoughtful comments regarding the limitations of the study. In response:

- We thank the reviewer for raising the issue of sample size. We agree that the size of the sample imposes certain limitations. However, the sample is sufficiently powered to identify overall trends and key group differences, which aligns with the main goal of the study. We have clarified this point in the revised discussion section.
- On the issue of disciplinary representation, we have included a more detailed reflection in the limitations section. While the final sample includes a higher proportion

of respondents from civil and environmental engineering and geography a lower proportion from biology and public health, a nonresponse analysis presented in Appendix A shows that only these two cases (civil engineering: overrepresented; biology and public health: underrepresented) were statistically significant, while the difference in representation for geography was not. We now note this explicitly in the discussion. However, we did not find relevant literature confirming systematic differences in early adoption patterns specifically for civil or environmental engineering or biology in the context of generative AI or technology. For this reason, and given the focus of our study, we chose not to expand further on disciplinary behavioral interpretations. That said, we have added a brief comment suggesting that these patterns might reflect different levels of exposure or familiarity.

- Finally, regarding the status of respondents (e.g., academic rank), we agree that this is an interesting and relevant dimension. Although we collected data on academic rank and verified that no significant bias existed in the sample composition, analyzing behavioral variation across rank—particularly in interaction with other variables such as gender or field—would require a more extensive statistical design. Without using multivariate techniques specifically tailored to detect such relationships, any interpretation would risk being misleading. Recognizing the value of this line of inquiry, we acknowledge that a detailed analysis of inter-group differences, such as behavioral variation across academic rank, gender, or field, is outside the scope of the current manuscript.

Changes in the text:

While this study provides valuable insights into the adoption intentions of academic scientists regarding generative AI, several limitations must be acknowledged. First, although based on probability sampling, the findings reflect responses from academic scientists across the six STEM disciplines included in the study, which may not represent the broader academic community. Additionally, as with any cross-sectional study, the findings reflect the period of data collection (late 2022–early 2023), capturing attitudes and experiences during the early adoption phase; perspectives may have since evolved. Smaller percentage differences should be understood within the survey's overall margin of error (±6.4%), though this does not affect the general interpretation of results, which point to trends in academic scientists' attitudes and experiences. Although no significant differences were observed in responses by gender or academic rank, the final sample does show variation in disciplinary representation. Civil and environmental engineering was more represented, while biology and public health were less so. These differences may reflect varying levels of exposure or familiarity with generative AI across disciplines.

Reviewer #5: Comment 1

As a first-time reviewer for a manuscript that has gone through a previous round of review, I have approached my task by looking only at the latest revised version of the manuscript, to see how it reads. And, in doing so, I find the manuscript to be generally tackling an interesting question, but with various weaknesses in the way it does so that make it unclear to me what, precisely, the authors are trying to achieve with their paper.

Authors' response:
Thank you very much for your work, time, and thoughtful comments on our manuscript. We truly appreciate your careful reading and the points you have raised. We would also like to note, in line with your own recognition, that the manuscript is at a stage where some of the current content and choices are the result of revisions made in response to comments from a previous round of review. We hope the reviewer will take this into consideration when reading the following responses, as it can sometimes be challenging to balance and integrate the differing suggestions and expectations of multiple reviewers. Nevertheless, we have made every effort to address all the feedback as thoroughly as possible.

Reviewer #5: Comment 2

1. The manuscript is - in parts - in need of copy-editing, as there are several grammatical errors or unnatural linguistic phrases/constructions. Having a native speaker take a (possibly closer) look at it to catch these issues would likely help. Personally, I also find the use of various "power-adjectives" to be not only (occasionally) over the top and less suited for the academic context (e.g. that AI has had a "transformative impact" or represents "a significant leap forward" - both highly contestable - and contested - claims), but also occasionally a bit too reminiscent of the sort of language that chatbots tend to produce. As much as I can appreciate the subtle meta-commentary of using occasional chatbot-like phrases in a paper on the use of chatbots in academia, if this is intentional I think it should be flagged a bit more transparently, and if it's accidental then it's effect is a bit of an unintentional (partial) undermining of the expected academic tone. Either way, I think it requires some reconsideration.

Authors' response:
Thank you for your comments. We would like to clarify that the authors are part of a diverse team, three of whom are native English speakers who have actively contributed to the writing and revision of the manuscript. Nevertheless, we have carefully considered the reviewer comments and have made further revisions to the manuscript, specifically aiming to remove or moderate the use of power-adjectives and value-laden expressions, in order to ensure a more objective and academically appropriate tone. We certainly do not want to leave any readers with the impression that chatbots of any sort might have participated in the preparation of this paper.

Reviewer #5: Comment 3

2. I find the literature review lacking. Both in the sense that there are what seem to me to be serious gaps in the referenced literature (e.g. not including Gebru's work on "stochastic parrots" - although it's both crucial to the area covered and has had a massive impact on the subsequent discussion - seems strange to me). But also in the sense that there seems to be two rather larger structural issues in the section.

Authors' response:
Thank you for your suggestion to include the influential work by Bender and Gebru (2021). We have now incorporated this reference into the introduction. We fully recognize the importance of engaging with such foundational contributions. However, we would also like to clarify that while our paper includes a literature review section to contextualize and justify the study, it is not intended as a comprehensive review of the broader field of generative AI. Our study is an empirical investigation specifically focused on perceptions and early adoption among faculty in research and education. Given the fast-paced evolution and complexity of the field, and with nearly 90 references already included, we acknowledge that some key works may not be covered. However, we believe the current scope of the literature review is sufficient for framing our research objectives, and further expansion would risk overcomplicating the paper without adding proportional value.

Changes in the text:

> *The ability of generative AI to produce text that is virtually indistinguishable from human-written work promises to transform educational methodologies and research practices, offering new opportunities for institutions, educators, and students [7,8]. This rapid expansion has also raised important questions regarding risks such as bias, transparency, and social impact [9].*

Reviewer #5: Comment 4

First, instead of discussing the benefits and risks of (gen)AI in academia, the authors go back and forth between the two, which makes for an awkward reading experience of "this sounds great, this sounds problematic, this sounds great, this sounds problematic". To be more concrete, the section has, after a brief introduction to AI in academia, a paragraph that starts with "However, despite perceived usefulness and improved ease of communication [33], there is insufficient understanding of generative AI's long-term impacts on educational practices...". This is then followed by a paragraph starting with "As generative AI continues to transform education, its impact is equally profound in the field of research, revolutionizing research by potentially acting as "an autonomous researcher," transforming the way knowledge is produced across the research process", which is problematic both in its repetition of terms such as "transformative" and in its overreliance on "power"-adjectives as per point 1 above. But this is then, AGAIN, followed by a paragraph introducing various problems, beginning with the sentence "While many of the applications of generative AI to

research activities appear promising, challenges and risks still loom large". This really needs some reconsideration of the structure of the themes and elements the authors wish to cover.

Authors' response:
We acknowledge the reviewer's observation regarding the "back-and-forth" between positive and negative aspects in the literature review, and we agree that this structure may have made the section somewhat difficult to follow in its original form. This impression is understandable, as the section was indeed designed to present a balanced view, highlighting both the benefits and the limitations of generative AI, across three key domains (education, research, and academic perceptions including survey-based studies). However, we also recognize that the lack of clearly defined subsections may have contributed to a sense of fragmentation. In response, we have now reorganized the literature review into distinct thematic subsections and have also made substantive changes throughout the entire review to improve clarity and coherence. We believe this revision significantly improves the structure, enhances readability, and helps guide the reader more effectively through the main arguments.

Reviewer #5: Comment 5

Second, and even more concerning, I don't find that the literature review section sufficiently sets up and qualifies the researchers' main focus, in the sense of clearly and unambiguously pointing to any important knowledge gaps. In the Introduction, the authors mention "Innovation Diffusion Theory", giving the impression this will constitute the theoretical framework for the remainder of the article, while the Lit Rev section presents this theory merely as one among others, with no indication as to why it constitutes a more relevant approach to their particular empirical undertaking. Further, the reliance on a rather broad range of references engenders some tension with the notion that the authors' own work has a sufficiently relevant value to warrant the research in the first place. I will say that I think this is primarily a writing issue: I believe that the research the authors have done is sufficiently important to warrant some attention and, ultimately, publication in a peer-reviewed journal. I just don't think that it is currently being set up in a sufficiently effective manner in the current version of their manuscript.

Authors' response:
We appreciate the reviewer's reflection on the effectiveness of the literature review to set up the empirical work in the paper. As we note in the paper, our focus is on the substantive gap in knowledge about early adoption of AI in academia. Given the focus, we believe the literature review provides sufficient backdrop to accomplish our exploratory and descriptive intentions. We have also made this focus clear at the beginning of the literature review to better guide the reader.

Changes in the text:

> *The following review is structured around the benefits and risks of generative AI in academia, setting the stage for an exploratory analysis of scientists' behaviors and perceptions.*

Reviewer #5: Comment 6

3. While I appreciate that the authors have based their work on the results of responses collected in '22 and '23, I think they need to more explicitly address the limitations this introduces, not least in terms of coming very short on the heels of the public launch of ChatGPT, meaning that attitudes today - in 2025, with all the far more wide-ranging experiences most of us have with genAI by now - might be significantly different. This is problematic in e.g. how the results are presented - phrasings such as "The data shows a majority engage with generative AI tools occasionally, reserving them for specific tasks rather than regular use" can be seen as potentially misleading - better replaced by introducing a qualifier such as "a majority during the studied time period" or similar - granted more clunky but at least more clearly indicating the actual knowledge that the responses allows one to surmise.

Authors' response:

We thank the reviewer for this suggestion. As with any cross-sectional study, our findings reflect the specific period in which the data were collected. While we acknowledge that attitudes and practices may have evolved since then, our focus on early adopters inherently situates the study in that initial phase of generative AI adoption in academia. To make this temporal framing more explicit, we have added a clarifying sentence to the Limitations section. We also believe the focus of the paper on the early-stage AI adoption in academia is a particular strength that deserves to be better highlighted.

Changes in the text:

> *First, although based on probability sampling, the findings reflect responses from academic scientists across the six STEM disciplines included in the study, which may not represent the broader academic community. Additionally, as with any cross-sectional study, the findings reflect the period of data collection (late 2022–early 2023), capturing attitudes and experiences during the early adoption phase; perspectives may have since evolved. Nevertheless, the findings of the paper on early-stage adoption behavior and perceptions provide a substantive and valuable basis for informing next stage research design and data collection.*

Reviewer #5: Comment 7

4. There are various issues with the statistics in the manuscript. For instance, the authors seek to assess "disciplinary variation" by performing a separate one-way ANOVA "across each response to each survey item (n=81 total comparisons)". They note that no potentially significant differences survived Bonferroni correction (.05 / 81). But given that some of their disciplines contain very few responders (e.g. 4 only in Public Health, 16 in Biology, 31 in Chemistry, etc.), this rather harsh correction for a rather absurdly large number of multiple comparisons cannot really be considered to allow the derivation of much of any relevant conclusion. I appreciate that this is one minor step - to remove concern that there could be significant hidden disciplinary variations among their respondents - but its application suggests to me a rather rudimentary appreciation of how to apply inferential statistics to real-world data. Essentially, with only 4 and 16 participants representing Public Health and Biology, respectively, a one-way ANOVA is ALREADY problematic, but to then further do an 81-comparison Bonferroni correction in order to suggest that there is no meaningful disciplinary variation in responses is such a stretch as to be generally irrelevant. Note also that this is but one example. To take another example: including four variations in phrasing of a key question introduces similar concerns, not least if cross-cutting with such categorizations as the respondents' field, rank, origin, etc. Basically, the small numbers of respondents found in each such categorization generally aren't large enough to permit much of any meaningful comparisons.

Authors' response:
We appreciate the reviewer's thoughtful comments and concerns regarding the statistical analyses. We would like to clarify several important points to contextualize the analysis in question.

First, although some disciplinary subgroups have small sample sizes, our dataset is weighted to improve representativeness across gender, academic field, and academic rank, as described in the methodology section and Appendix A. Additionally, we conducted non-response bias analyses, which confirmed that the final sample is largely representative of the broader population of academic scientists at R1 institutions across the selected disciplines.

Second, the one-way ANOVA and the subsequent Bonferroni correction were both included during the first round of peer review, in response to a prior reviewer's request to explore whether disciplinary differences meaningfully shaped responses. While we recognize the limitations of statistical power, particularly due to small sample sizes in certain fields, the analysis was included as a supplementary and exploratory step, not to make strong inferential claims.

Third, the Bonferroni correction, while conservative, was applied to transparently address the risk of false positives across multiple comparisons. We acknowledge this approach may limit sensitivity to subtle effects, but we consider it the most transparent and cautious way to address the prior reviewer's concern.

However, after careful consideration of the current reviewer's feedback, and in recognition that the inclusion of this analysis raises more questions than it resolves, we have decided to remove this analysis from the manuscript. We appreciate that the reviewer has highlighted its limitations, which we have explicitly acknowledged in the manuscript.

Reviewer #5: Comment 8

5. The fact that there are essentially only descriptive (mostly frequency) statistics here are surprising to me. As much as I am of the opinion that researchers ought to do more exploratory analysis in general (as per Tukey), this ought typically to be in the service of either grounding subsequent inferential analysis or - perhaps - generating hypotheses for further (future) inferential testing. If the authors see themselves as falling with the exploratory-data-analysis camp, this is laudable and valuable, but then it needs to be more explicitly flagged as such throughout. In its current state, the manuscript reads more like a rather straightforward confirmatory-statistics paper, introducing aims, testing them, and drawing conclusions on the basis of the results found. But if the authors do see themselves as performing more confirmatory-style hypothesis-testing, then they would need to explore both descriptive and inferential statistics to a much greater degree (e.g. through basic measures of central tendency and variability, and inferential tools such as linear mixed models or whatever else the authors consider most appropriate), and further include statistical power calculations and the like, given their limited number of respondents. In general, I'd even go so far as to say that the repeated use of the term "analysis" isn't quite warranted by the actual research, which performs little actual analysis beyond presenting frequencies (percentages) of forced-choice responses to survey items. In this last respect, much of the manuscript findings read more like an interesting news story than a full-fledged research analysis of collected data.

Authors' response:

We appreciate the reviewer's reflection on the nature and purpose of our analysis. We confirm that the study is designed as a descriptive and exploratory analysis of academic scientists' reported perceptions, experiences, and uses of generative AI during an early stage of adoption. Our primary aim is to present a structured, representative overview of attitudes and behaviors rather than to test formal hypotheses or establish causal inference. While the manuscript includes clearly defined research aims and uses the term "analysis" throughout, we believe these are not incompatible with an exploratory-descriptive approach. In fact, clearly articulated objectives and systematic data analysis are essential components of rigorous descriptive research. However, we acknowledge that the manuscript could better signal this methodological positioning. To address this, we have now explicitly stated the descriptive and exploratory nature of the study in the introduction.

Changes in the text:

> *To achieve these insights, we draw from a nationally representative survey of academic scientists, that include assistant, associate and full professors, across six disciplines—biology, chemistry, computer and information science engineering, civil*

and environmental engineering, geography, and public health—at randomly selected Carnegie-designated Research Extensive and Intensive (R1) universities. The study is designed as a descriptive and exploratory analysis of academic scientists' reported attitudes, experiences, and uses of generative AI. The following aims were established to effectively address this main objective:
