# Supplementary material for: Generative AI and academic scientists in US universities: Perception, experience, and adoption intentions
Source: PLoS One. 2025 Aug 28;20(8):e0330416. doi: 10.1371/journal.pone.0330416 (PMC12393709; doi:10.1371/journal.pone.0330416)
Supplement: Attachment — Submitted filename: review_plosone.docx [file pone.0330416.s004.docx]

The manuscript presents an insightful study on the integration of Generative AI in academia, focusing on US academic scientists’ adoption, perceptions, and regulatory concerns. The topic is highly relevant, and the study provides valuable data from a nationally representative survey of STEM faculty. The findings contribute to ongoing discussions on AI’s role in education and research. However, certain areas require further clarification and expansion, particularly regarding methodology, theoretical grounding, and the discussion of ethical concerns. Below are specific strengths and areas for improvement.

Areas for Improvement:

- The manuscript would benefit from engagement with established technology adoption models, such as the Technology Acceptance Model (TAM) or Unified Theory of Acceptance and Use of Technology (UTAUT).
- Integrating these frameworks would help contextualize the faculty’s perceptions and concerns regarding AI adoption.
- While the study is based on a nationally representative survey, key methodological details are missing, including:

Sampling technique and representativeness of different institution types.

Survey response rate and potential response bias.

Validation of survey questions to ensure reliability and relevance.

Providing more transparency in these areas would enhance the study’s rigor.

- While the study mentions misinformation concerns (78%), it could also discuss other ethical implications, such as:

Bias in AI models and how it affects academic use.

Data privacy issues in AI-generated content.

Plagiarism detection challenges in AI-assisted research.

- A deeper engagement with these ethical dimensions would strengthen the manuscript’s contribution.
- The study focuses on STEM faculty, which may limit generalizability to the humanities, social sciences, and international academic institutions.
- Expanding the discussion on how AI adoption may differ across disciplines would enhance the broader relevance of the findings.
- While the study states that faculty support academic-led AI regulation, it does not provide concrete policy recommendations or engagement with existing AI governance frameworks.
- Expanding this discussion with references to current AI policies in education would improve the impact of the study’s findings.
